# Comorbidity factors between Obsessional-Compulsive Disorder (OCD) and Schizophrenia: Through Case Report and Literature Review

**DOI:** 10.1192/j.eurpsy.2025.1197

**Published:** 2025-08-26

**Authors:** A. Daoud, N. Halouani, S. Elloumi, C. Walha, S. Ellouze, M. Turki, J. Aloulou

**Affiliations:** 1 Psychiatry B, Hospital University Hedi Chaker, Sfax, Tunisia

## Abstract

**Introduction:**

Obsessional-Compulsive Disorder (OCD) can involve poor insight, which may complicate the differential diagnosis of schizophrenia. Otherwise, few cases may associate both diagnosis highlighting the need for a clearer understanding of the factors of their coexistence.

**Objectives:**

Understand the factors that contribute to the comorbidity between OCD and schizophrenia.

**Methods:**

Our study employed a case report approach that describes a patient admitted in our psychiatric unit. Data were collected through clinical interviews, personal and family medical history. We also conducted a literature research focusing on articles published between 2000 and 2023 in PubMed using the keywords « OCD », « schizophrenia » and « comorbidity ».

**Results:**

The case concerns Mr. N, a 43-year-old man with no significant medical or psychiatric history and no reported substance use behaviors, who was admitted to our psychiatric unit. He was diagnosed with both OCD and schizophrenia. The therapeutic evaluation revealed that Mr. N was initially prescribed anxiolytics, with a second-generation antipsychotic subsequently added. Some researches suggests that second-generation antipsychotics may induce obsessive-compulsive symptoms which could explain the coexistence of the two diagnosis in Mr N. Additionally, other studies have indicated that genetic factors can predispose individuals to both conditions whereas our patient does not have any notable family history of psychiatric disorders. Finally, literature says that neurobiological factors particularly shared dopaminergic and serotonergic pathways may provide an explanation for the comorbidity of OCD and Schizophrenia.

**Conclusions:**

The comorbidity of OCD and schizophrenia may be explained by several factors, making diagnosis and treatment challenging.

**Disclosure of Interest:**

None Declared

